# Dynamic association of the H3K64 trimethylation mark with genes encoding exported proteins in *Plasmodium falciparum*

**DOI:** 10.1016/j.jbc.2021.100614

**Published:** 2021-04-09

**Authors:** C.A. Jabeena, Gayathri Govindaraju, Mukul Rawat, Soundhararajan Gopi, Devadathan Valiyamangalath Sethumadhavan, Abdul Jaleel, Dhakshmi Sasankan, Krishanpal Karmodiya, Arumugam Rajavelu

**Affiliations:** 1 Pathogen Biology Group, Rajiv Gandhi Centre for Biotechnology (RGCB), Thiruvananthapuram, Kerala, India; 2 Manipal Academy of Higher Education (MAHE), Manipal, Karnataka, India; 3 Department of Biology, Indian Institute of Science Education and Research, Pune, Maharashtra, India; 4 Department of Biotechnology, Bhupat & Jyoti Mehta School of Biosciences, Indian Institute of Technology Madras, Chennai, India; 5 Cardiovascular Disease Biology, Rajiv Gandhi Centre for Biotechnology (RGCB), Thiruvananthapuram, Kerala, India

**Keywords:** Epigenetics, malaria, histone methylation, exported family proteins, nucleosome, ChIP, chromatin immunoprecipitation, H3K64, histone 3 at lysine 64, H3K64me3, trimethylation at lysine 64 on histone 3, IDC, intra-erythrocytic developmental cycle, PDB, Protein Data Bank, PfSET, *Plasmodium falciparum* Su (*var*) 3-9, Enhancer, RBCs, red blood cells, SET, Su (*var*) 3-9, Enhancer-of-zeste, and Trithorax

## Abstract

Epigenetic modifications have emerged as critical regulators of virulence genes and stage-specific gene expression in *Plasmodium falciparum*. However, the specific roles of histone core epigenetic modifications in regulating the stage-specific gene expression are not well understood. In this study, we report an unconventional trimethylation at lysine 64 on histone 3 (H3K64me3) and characterize its functional relevance in *P. falciparum*. We show that PfSET4 and PfSET5 proteins of *P. falciparum* methylate H3K64 and that they prefer the nucleosome as a substrate over free histone 3 proteins. Structural analysis of PfSET5 revealed that it interacts with the nucleosome as a dimer. The H3K64me3 mark is dynamic, being enriched in the ring and trophozoite stages and drastically reduced in the schizont stages. Stage-specific global chromatin immunoprecipitation –sequencing analysis of the H3K64me3 mark revealed the selective enrichment of this methyl mark on the genes of exported family proteins in the ring and trophozoite stages and a significant reduction of the same in the schizont stages. Collectively, our data identify a novel epigenetic mark that is associated with the subset of genes encoding for exported proteins, which may regulate their expression in different stages of *P. falciparum.*

*Plasmodium falciparum* is the most severe and prevalent malaria parasite, and about 228 million malaria cases were reported in 2018 (https://www.who.int/malaria/publications/world_malaria_report/en/). With increased investments in malaria elimination programs and therapeutic interventions, the worldwide malaria burden started declining. However, the emergence of artemisinin-resistant *P. falciparum* toward most currently available drugs including artemisinin-based combination therapies is of great concern (1, 2). Five major species of *Plasmodium* that can infect humans are *P. falciparum*, *Plasmodium vivax*, *Plasmodium ovale, Plasmodium malariae,* and *Plasmodium knowlesi*, of which the *P. falciparum* is the most virulent species that causes cerebral malaria with high chances of mortality (3, 4). The *P. falciparum* is an obligate intracellular parasite and has a complicated life cycle. Asexual development occurs inside the red blood cells (RBCs) of humans where the parasite develops through the ring, trophozoite, and multinucleated schizont stages and ruptures the RBCs to release up to 30 merozoites that can further invade new RBCs and continue their development (5). The parasite uses various regulatory mechanisms for the completion of its life cycle to survive inside RBCs. The gene regulatory mechanisms are regulated in an intriguing and complex way as the *P. falciparum* adapts to the multiple stages during its development. Recent reports highlight the importance of epigenetic players in controlling the chromatin organization, gene regulation, morphological differentiation, and antigenic variation in *P. falciparum* (6, 7, 8).

The epigenome of *P. falciparum* is distinct from mammals as it lacks the linker histone protein H1, diverse set of transcription factors, and canonical RNA interference machinery (9, 10, 11). The DNA methylation is poorly characterized in the malaria parasite, reports had shown the presence of residual DNA methylation in *P. falciparum* (12, 13), and a recent report has identified the presence of 5hmC-like modification in the genome of the *P. falciparum* (14). However, another study has reported that DNMT2, a DNA methyltransferase homolog in *P. falciparum* 3D7 preferably methylate the tRNA aspartic acid (15). Thus, it implies that the chromatin modifications and nucleosome dynamics are the key in the regulation of stage-specific differential gene expression in *P. falciparum* (16, 17).

The differences in nucleosome dynamics contribute to the chromatin structures that are associated with the transcriptional activity of several invasion genes (17). It was proposed that extremely high AT content (81%) in the genome can affect the proper positioning of nucleosomes in *P. falciparum* (16). However, reports have shown that nucleosome dynamics of the parasite in RBC stages are not majorly mediated by AT-rich genome or through histone variants (18, 19). This strongly implies that the formation of functional chromatin structure may require extranucleosomal positioning signals viz locus-specific methylation, acetylation on the histone proteins to achieve the successful chromatin organization in *P. falciparum* (20, 21). The dynamic distribution of methyl marks and its functional readout through epigenetic methyl binding proteins are essential to maintain the proper chromatin structure as well as to regulate the precise gene expression. It is known that “histone tail” modifications are essential in recruiting effector proteins, whereas “histone core” modifications are essentially involved in the regulation of nucleosome dynamics (22). The globular domain methylations of histone proteins viz H3K56, H3R42, H3K79, H4K91, and histone 3 at lysine 64 (H3K64) are known to play major roles in gene suppression in mammals (23, 24).

Reports had shown that the trimethylation at lysine 64 on histone 3 (H3K64me3) mark is enriched in the pericentric heterochromatin, repeat sequences, and transcriptionally repressed regions (24, 25). In humans, unlike histone tail modifications, which regulate the gene expression at the DNA level, the globular domain modifications help in nucleosome dynamics and thereby modulating chromatin plasticity (22). The previous reports have shown that epigenetic modifications are involved in stage-specific gene expression in *P. falciparum* (6, 7). However, the role of histone core modifications in not studied so far.

Here, we report on the histone lysine methylation mark on the lateral surface of H3K64 in *P. falciparum*. Using various biochemical assays, we have identified that PfSET4 and PfSET5 enzymes methylate at H3K64 with PfSET5, showing more activity toward the 64th lysine of the histone H3 subunit than PfSET4 protein. Both the enzymes prefer nucleosome as a substrate and do not methylate free histone 3 protein. The structural characterization of PfSET5 has identified that the enzyme forms the dimer to recognize nucleosome substrates. We found that the H3K64me3 mark is enriched in the ring and trophozoite stages and reduced in the multinucleated schizont stages of *P. falciparum*. The global chromatin immunoprecipitation (ChIP)-sequencing analysis has identified that the H3K64me3 mark is reduced on the genes of exported proteins in the schizont stage of *P. falciparum*. We observed an inverse correlation of expression of the exported family of proteins to the deposition of the H3K64me3 mark in the RBC stages of *P. falciparum*. Taken together, our results provide the first evidence on the dynamic association of the H3K64me3 mark with the subsets of genes that encode for exported proteins and might potentially involve in regulation of gene expression in *P. falciparum*.

## Results

### Identification of K64 trimethylation in histone 3 of *P. falciparum*

**Figure 1:**
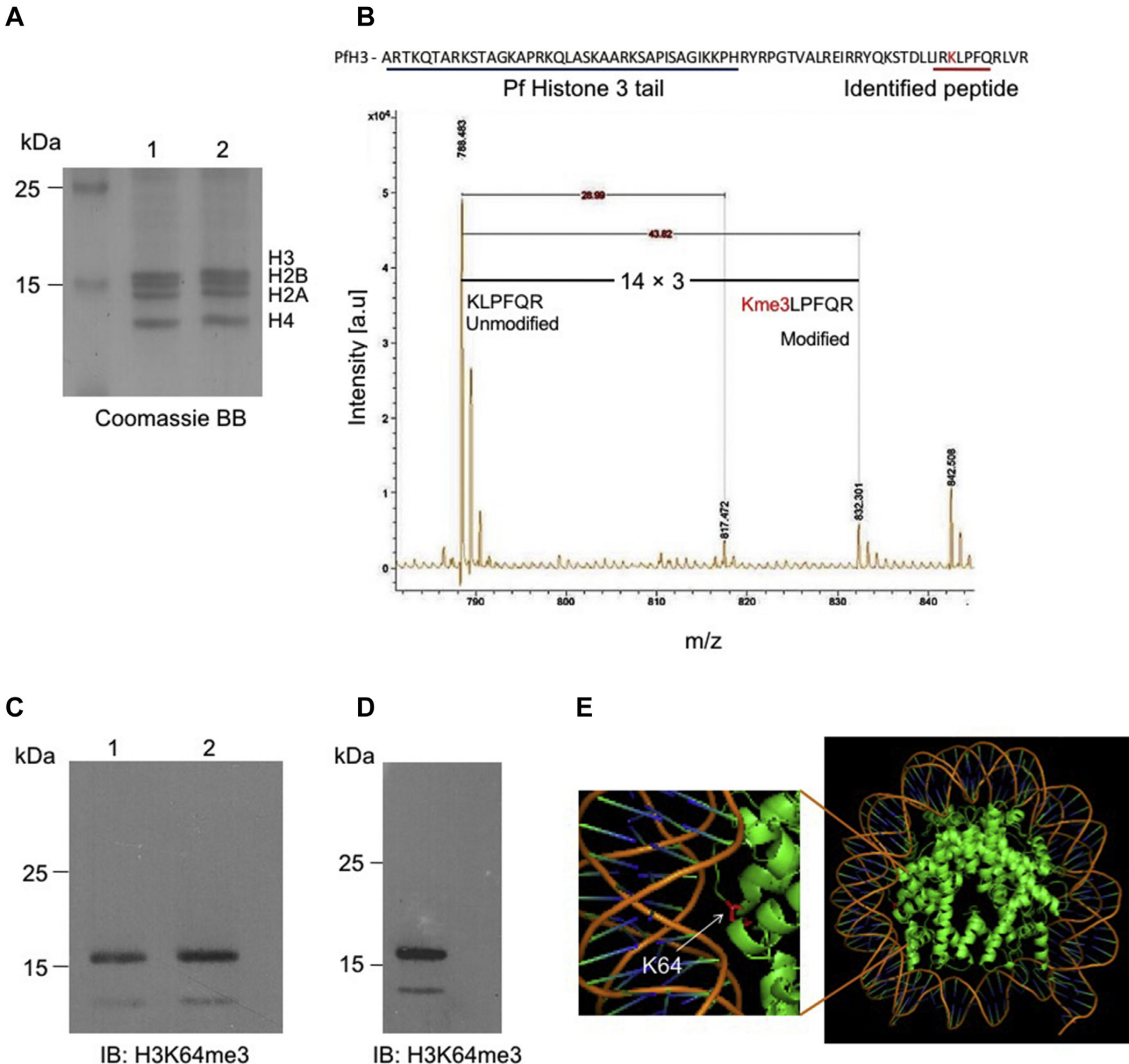
**Identification of H3K64me3 mark in *P. falciparum*.***A*, two independent histone preparations from the *P. falciparum* trophozoite stage were separated on 16% SDS-PAGE gel and stained with Coomassie Brilliant Blue, and the major four histone proteins of *P. falciparum* are labeled. *B*, the sequence of *P. falciparum* H3 protein; the *blue underline* represents the N-terminal tail portion of the H3 subunit and *red underline* represents identified peptides from MALDI-TOF and the target lysine 64 is marked in *red*. At the bottom, the MALDI-TOF spectrum of H3-specific peptide with 43-Da differences of modified and unmodified peptide mass is represented (Fig. S1 for fragmentation spectra). *C*, the immunoblot analysis of two independent histone preparations from *P. falciparum* with the anti-H3K64me3 antibody identified the presence of methyl mark on the H3 subunit matching to 16 kDa. *D*, immunoblot analysis using the anti-H3K64me3 antibody for the histone protein isolated from a clinical isolate of *P. falciparum*. *E*, the positioning of K64 on the histone 3 subunit in the mononucleosome structure. The expanded structure represents the target lysine (K64), marked in *pink*, located at the lateral surface of core nucleosome and very proximal to the DNA contact point. H3K64me3, trimethylation at lysine 64 on histone 3.

The unique methylation marks on the H3 subunit were identified in the acid-extracted histone proteins (H3, H2B, H2A, and H4) isolated from the trophozoite stage of parasite culture and separated on 16% SDS-PAGE (Fig. 1*A*). The Coomassie Blue–stained gel band corresponding to the H3 subunit (around 16 kDa) was excised and subjected to MS analysis. Subsequently, the acquired raw data were subjected to database search using MASCOT for protein identification. Mass spectrum of the peptides shows the mass shift of 43 Da in the modified peptide, which further validated the presence of trimethyl groups (Figs. 1*B* and S1). The *P. falciparum* encodes H3 protein and its variant H3.3, of which the variant H3.3 demarcates euchromatic coding and subtelomeric regions of *P. falciparum* (26). The identified H3K64me3 modified peptide matches to both H3 and H3.3 proteins, and the present study focus only on H3. The immunoblot analysis of histone proteins extracted from *P. falciparum* with anti-H3K64me3 antibody further confirmed the presence of the methyl mark on the H3 subunit of the parasite (Fig. 1*C*).

The specificity of the H3K64me3 mark antibody was verified using a dot blot assay with synthetic peptides containing various methylation marks (Fig. S2; Supplementary File 1, sheet 6). To rule out the possibility of *in vitro* culture–adapted acquisition of the H3K64me3 mark, we validated the presence of the H3K64me3 mark on histone proteins extracted from fresh clinical isolates of *P. falciparum* (Fig. 1*D*), which strongly suggests the physiological relevance of the H3K64me3 mark in *P. falciparum*. To locate the H3K64me3 position in the nucleosome, we have analyzed the human nucleosome structure (Protein Data Bank [PDB] # 2CV5) and found that lysine 64 (K64) is located on the lateral surface of the H3 subunit and resides near to the DNA contact point (Fig. 1*E*). It is reported that the structure of the nucleosome in *P. falciparum* is found to be dynamic throughout its development inside the RBCs (17), thus the possibility of trimethylation at H3K64 may directly or indirectly contribute to this process as the methyl mark resides very close to the DNA contact point in the nucleosome.

### Identification of histone lysine methyltransferases for H3K64 from *P. falciparum*

**Figure 2:**
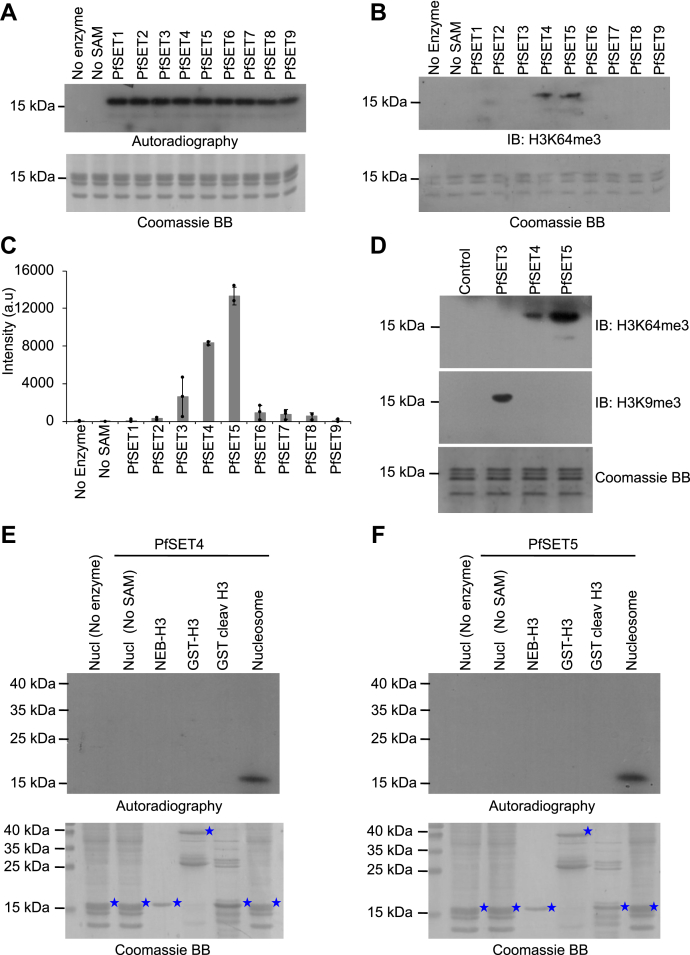
**Identification and characterization of H3K64 histone lysine methyltransferases from *P. falciparum*.***A*, *in vitro* methyltransferase activity of 9 PfSET domain recombinant proteins of *P. falciparum* using tritium-labeled SAM as cofactor on the mononucleosome substrates, and all the 9 PfSET enzymes were active on the nucleosome substrate. The *bottom lane* represents the loading control of nucleosome substrate separated on 16% SDS-PAGE gel stained with Coomassie Brilliant *Blue*. No enzyme and no SAM controls were included in the assay. *B*, *in vitro* methylation activity of 9 PfSET domain proteins on the synthetic mononucleosome substrate using nonradiolabeled SAM as a cofactor, followed by separation of samples on 16% SDS-PAGE gel, transferred and probed with the anti-H3K64me3 antibody. The *bottom image* represents the loading control of the synthetic mononucleosomes stained with Coomassie Brilliant *Blue* stain. *C*, the intensity of the immunoblot image from *B* was analyzed using ImageJ software and plotted against intensity, PfSET5 and PfSET4 showed significant methyltransferase activity toward H3K64. The error bar represents the SD of three biological replicates. *D*, *in vitro* methylation activity of PfSET3, PfSET4, and PfSET5 proteins on the synthetic mononucleosomes substrate using nonradiolabeled SAM as a cofactor, followed by immunoblot with anti-H3K64me3 and anti-H3K9me3 antibodies. The *bottom image* represents the loading control of the synthetic mononucleosomes stained with Coomassie Brilliant *Blue*. *E* and *F*, *in vitro* methylation activity of PfSET4 and PfSET5 proteins on the recombinant histone 3 (HsH3) proteins (NEB company), recombinant GST-tagged PfH3 proteins, GST-cleaved PfH3 proteins, and mononucleosomes substrates using radiolabeled SAM as cofactor and no enzyme and no SAM controls were included for both proteins. The *bottom image* shows the loading control of all three histone proteins and mononucleosomes separated on 16% SDS-PAGE gel and stained with Coomassie Brilliant *Blue* stain. The histone 3 proteins are marked with *blue stars*. H3K64, histone 3 at lysine 64; H3K64me3, trimethylation at lysine 64 on histone 3.

The earlier report identified that H3K64me3 functions as a repressor methyl mark in mammals (24, 25). However, the H3K64me3-specific methyltransferases are not identified to date in any of the eukaryotes, but it was reported that knockdown of Suv39H1, a well-known H3K9 methyltransferase, leads to the reduction of the H3K64me3 mark and proposed that Suv39H1 could methylate both H3K9 and H3K64 in mammals because of conservation of the RK motif in both positions on the H3 substrate (24). To identify the H3K64 methyltransferases in the malaria parasite, we cloned, expressed, and purified 9 SET (Su (*var*) 3-9, Enhancer-of-zeste, and Trithorax) domains from *P. falciparum* as glutathione-*S*-transferase (GST)-tagged recombinant proteins (Fig. S3, *A* and *B*). We tested the enzymatic activity using tritiated SAM as a cofactor and found that all the 9 SET domains were active on *P. falciparum* nucleosome substrates (Fig. 2*A*). Although all the purified *Plasmodium falciparum* Su (*var*) 3-9, Enhancer (PfSET) enzymes are active on the mononucleosome, this radiolabeling methylation assay would not provide the exact target lysine that gets methylated on histone proteins. To identify the PfSET enzymes that methylate at the H3K64 position, we used synthetic mononucleosome without any post-translational modifications for enzyme assays (EpiCypher) and the quality of the nucleosome was verified using 16% SDS-PAGE gel followed by Coomassie Blue staining (Fig. S3*C*). The methylation assay was performed with PfSET enzymes using nonradiolabeled SAM as a cofactor followed by immunoblotting with the anti-H3K64me3 antibody. The results have identified that PfSET4 and PfSET5 preferentially methylated at the H3K64 position (Fig. 2, *B* and *C*), of which the PfSET5 enzyme shows strong activity toward H3K64 when compared with the PfSET4 enzyme. We also observed a weak signal with PfSET3-methylated nucleosome, which is known to methylate at H3K9 in *P. falciparum* (27). This may be due to the conservation of the RK motif at K9 and K64 positions in the histone 3 proteins (Fig. S4).

Furthermore, we performed the methylation assay for PfSET4 and PfSET5 enzymes with the synthetic mononucleosomes and immunoblotted with the anti-H3K9me3 antibody. We observed that both enzymes do not methylate at the H3K9 position, rather they methylate at 64th lysine residue of the H3 subunit (Fig. 2*D*). Also, we have identified that the PfSET3 enzyme methylates only H3K9 and does not methylate H3K64, which suggest that PfSET4 and PfSET5 are the two enzymes evolved to methylate a target lysine (64th lysine residue of H3) and do not have the cross enzymatic activity to H3K9 residue of H3 in *P. falciparum* (Fig. 2*D*). As H3K64 resides very proximal to the DNA contact in the nucleosome, we tested the substrate selectivity of PfSET4 and PfSET5 enzymes. The radiolabeling assays on various substrates have clearly shown that both PfSET4 and PfSET5 enzymes selectively methylate nucleosome substrates, but do not methylate free histone substrates (Fig. 2, *E* and *F*) (Fig. S5). This suggests that H3K64 methyltransferases prefer an intact nucleosome as the substrate to successfully methylate the target K64 on histone 3 subunits in *P. falciparum*.

### PfSET4 and PfSET5 enzymes methylate at H3K64

**Figure 3:**
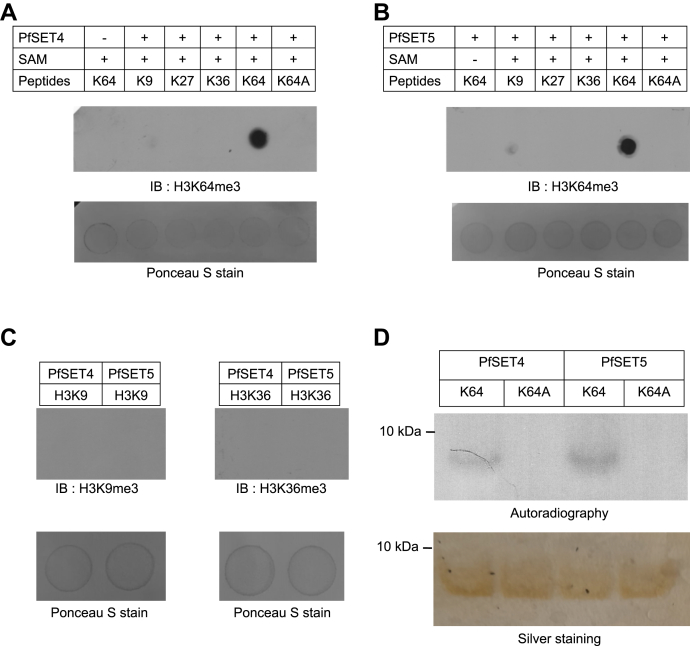
**PfSET4 and PfSET5 proteins methylate at H3K64.***A* and *B*, the *dot blot* assay with PfSET4 and PfSET5 enzymes on various synthetic peptides from H3 flanking potential target lysine viz H3K9, H3K27, H3K36, and H3K64 and H3K64A mutant peptides. No enzyme and no SAM controls were included in the membranes. The methylated peptides were spotted on nylon membrane and immunoblot was carried using the anti-H3K64me3 antibody. *Bottomimages* represent the Ponceau S staining of peptides on the membrane collected before blocking with skim milk powder. *C*, dot blot assay for PfSET4 and PfSET5 enzymes with H3K9 and H3K36 peptides, followed by immunoblotting with anti-H3K9me3 and anti-H3K36me3 antibodies to rule out the absence of methylation activity by these two enzymes on these peptides. *Bottom* images represent the Ponceau S staining of peptides on the membrane collected before blocking with skim milk powder. *D*, *in vitro* methylation assay using tritiated SAM for H3K64 WT and H3K64A mutant peptides with PfSET4 and PfSET5 proteins. The samples were separated on any kDa PAGE gel, dried, and exposed to X-ray film. The *bottom* gel image represents the loading control samples separated on any kDa PAGE gel, followed by silver staining indicating the equal loading of the K64 WT and K64A mutant peptides. The peptide methylation assays were performed in three biological replicates, and the representative images from the single assay is provided. H3K64, histone 3 at lysine 64.

The PfSET4 and PfSET5 enzyme activities were verified using various synthetic histone peptides (Supplementary File 2, sheet 6). *In vitro* peptide methylation assay for various target lysine peptides (H3K9, H3K27, H3K36, H3K64, and H3K64A peptides) with PfSET4 and PfSET5 enzymes followed by the dot blot assay with the anti-H3K64me3 antibody was performed. The results showed that both enzymes prefer to methylate at H3K64 peptides (Fig. 3, *A* and *B*). In addition, we performed methylation with H3K9 and H3K36 peptides for PfSET4 and PfSET5 enzymes, followed by dot blot with anti-H3K9me3 and anti-H3K36me3 antibodies, which revealed that these enzymes do not prefer to methylate at H3K9 and H3K36 peptides (Fig. 3*C*). To substantiate our results, we performed a methylation assay for both proteins with H3K64 WT and H3K64A mutant peptides using tritiated SAM as a cofactor, and the methylated peptides were separated on any kD PAGE gel. The autoradiography image confirms that PfSET4 and PfSET5 enzymes methylate at H3K64 and mutating the K64 to alanine in the substrate peptide leads to complete reduction of the enzyme activity by PfSET4 and PfSET5 enzymes (Fig. 3*D*). The possible reason for methylation of the PfSET4 and PfSET5 enzymes on the peptides rather than histone proteins might be due to the small size and easy accessibility of the peptides to the active sites of these enzymes. Thus, the biochemical assay confirms that the PfSET4 and PfSET5 enzymes methylate at H3K64 and do not methylate at K9 and K36 on histone 3 proteins.

### PfSET5 recognizes the nucleosome to methylate at H3K64

**Figure 4:**
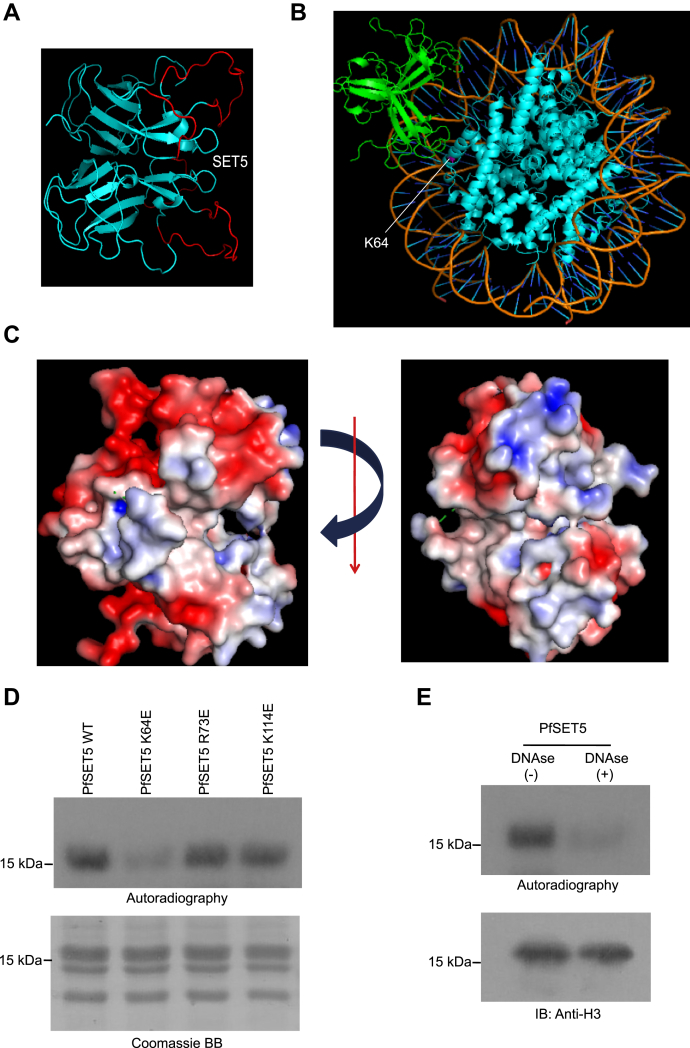
**Structural analysis of PfSET5 protein.***A*, the X-ray crystal structure of PfSET5 (PDB # 4RZ0) protein shows it forms a dimer, and the missing loops in the structure were filled using MODELLER to obtain the complete protein structure. The filled missing loops from the original structure are shown in *red*. *B*, molecular docking analysis of PfSET5 protein with mononucleosome and the PfSET5 dimer protein interacts with mononucleosome on the DNA molecule; the target lysine (H3K64) is marked in *pink*. *C*, surface potential analysis of PfSET5 protein revealed that the dimer protein forms basic amino acids patches (*blue*) through which the protein interacts with the DNA structure on the nucleosome. The *red* patches are acidic amino acids on the protein surface. *D*, *in vitro* methyltransferase assay with PfSET5 WT and mutant proteins (PfSET5 K64E, PfSET5 R73E, and PfSET5 K114E) on the nucleosome substrate using radiolabeled SAM as a cofactor. *E*, *in vitro* methyltransferase assay for PfSET5 WT protein with DNase-treated and DNase-untreated mononucleosome substrates using radiolabelled SAM as a cofactor. The samples are separated on 16% SDS-PAGE gel, dried, and exposed to the X-ray film. The *bottom* immunoblot image represents that intact H3 levels in both DNase-treated and DNase-untreated samples that rule potential carryover of protease contamination. PDB, Protein Data Bank.

The nucleosome-specific activity of PfSET4 and PfSET5 on H3K64 is exciting as these two proteins do not methylate free histone protein. Particularly, it is interesting to note that PfSET5 is a small protein consisting of 178 amino acids that harbor an SET domain and does not contain any regulatory domains (Fig. S3*A*). To address the mechanisms of nucleosome specificity by PfSET5, we used the X-ray crystal structure deposited by Structural Genomics Consortium, and the resolution of the X-ray crystal structure of PfSET5 is 2.4 Å (PDB # 4RZ0) (28). The available PfSET5 crystal structure in the PDB portal lacks significant loops in the structure, and the missing loops in the protein were modeled as described in the Experimental procedures section to generate the complete structure of PfSET5 protein. The structural data show that PfSET5 forms a dimer with two monomer subunits facing opposite directions (Fig. 4*A*).

To get mechanistic insights on how the PfSET5 dimer protein interacts with the nucleosome substrate, we performed molecular docking analysis of PfSET5 with nucleosome structure to identify the potential interface on PfSET5 that mediates its interaction with the nucleosome. The molecular docking analysis has shown that PfSET5 interacts with DNA on the nucleosome using both monomer subunits (Fig. 4*B*). The surface potential analysis has identified that the PfSET5 dimer forms basic amino acid patches that mediate the interaction of the protein to the negatively charged DNA on the nucleosome (Fig. 4*C*). The amino acids K64, R73, and K114 of PfSET5 protein are the key three basic amino acids that form basic patches to interact with the negatively charged DNA structure on nucleosome (Fig. 4*C*). We hypothesized that mutating the positively charged amino acids to negatively charged amino acids in PfSET5 could reduce the protein interaction with the negatively charged nucleosome structure. To test this, we have mutated the lysine and arginine amino acids in the basic patches to glutamic acid in PfSET5, which confirmed the presence of the right mutation by Sanger sequencing. The PfSET5 mutant proteins were expressed and purified with the quality equivalent to that of the WT protein (Fig. S6). The methylation assay with the nucleosome using mutant PfSET5 enzymes revealed a significant reduction in methylation activity of PfSET5 K64E mutant enzyme on the nucleosome substrate (Fig. 4*D*). The other two mutant proteins did not show any changes with methyltransferase activity (Fig. 4*D*). This suggests the K64 in PfSET5 proteins might involve in the interaction with the nucleosome substrate.

It is possible that upon binding to the nucleosome substrate, the PfSET5 protein may undergo conformational changes to reach the target lysine at the 64th position of the H3 subunit. The structural changes could be potentially due to the reorganization of the disordered charged loops missing in the crystal structure (residues 57–76 and 104–113 in 4RZ0) harboring K64 that display a drastic reduction in methylation upon charge reversal mutation. To address this, we performed a methylation assay with DNase-treated nucleosome as the substrate that removes the DNA from the core nucleosome and performed methylation assay with WT PfSET5 enzyme. We observed reduced methylation activity of PfSET5 enzyme on DNase-treated mononucleosome than with the undigested mononucleosome substrates, which strongly suggest that intact nucleosome and hence the negative potential around the DNA is essential to methylate at H3K64 by PfSET5 WT enzyme (Fig. 4*E*). Overall, our results identified two enzymes PfSET4 and PfSET5 from *P. falciparum* methylate at H3K64 on the nucleosome substrates.

### Dynamic distribution of the H3K64me3 mark in RBC stages of *P. falciparum*

**Figure 5:**
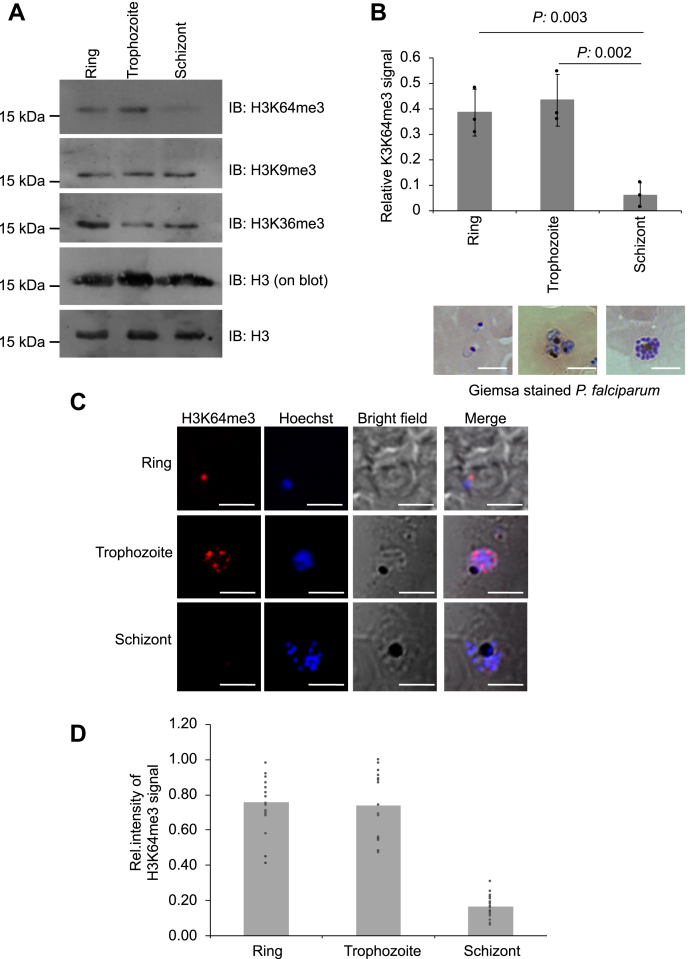
**Dynamics H3K64me3 mark during RBC stages of *P. falciparum.****A*, immunoblot analysis of histone proteins extracted from a synchronous culture of *P. falciparum,* and a strong signal was detected in ring and trophozoite stages for H3K64me3 modification. A well-known gene repressor methyl marks viz H3K9me3 and H3K36me3 on H3 protein were used as a control which remained the same throughout the RBC stages. Two separate H3 protein loading controls were used: (i) H3K64me3 blot was stripped and probed with the anti-H3 antibody (on *blot*), (ii) independent loading of the same concentration of histone proteins and probed with the anti-H3 specific antibody (*lower panel blot*). *B*, the relative intensity of H3K64me3 signal obtained using ImageJ and normalized to H3 loading control (on *blot*). The bar diagram was prepared from average values from three independent biological replicates, the error bar represents the SD of three biological replicates, and the *p*-values are calculated with individual paired *t* test calculation that shows a significant reduction in the H3K64me3 intensity in schizont stages of *P. falciparum*. The *bottom* images are Giemsa-stained representative images of the synchronous culture of *P. falciparum* at three stages: ring, trophozoite, and schizont. The scale bar represents 5 μ. *C*, immunofluorescence-stained representative images from three stages of *P. falciparum* with the anti-H3K64me3 antibody followed by the secondary antibody, and nuclear staining was performed with Hoechst staining. The scale bar represents 5 μ; additional images are provided in the Figure S9. *D*, the bar diagram represents the average relative intensity of H3K64me3 to DAPI signal stain in *P. falciparum* parasite, which was calculated using ImageJ software; each distribution point represents single parasite from each stage (n =21). H3K64, histone 3 at lysine 64; H3K64me3, trimethylation at lysine 64 on histone 3; RBCs, *red* blood cells.

The chromatin architecture of *P. falciparum* is dynamic throughout its intra-erythrocytic developmental cycle (IDC) stages, which may regulate the stage-specific differential gene expression that helps the parasite to develop and survive inside the host. Because the H3K64 resides at the DNA contact point of the histone, we hypothesized that this unconventional methyl mark could be dynamically deposited in various developmental stages of *P. falciparum.* To address this, we examined the levels of the H3K64me3 mark on the H3 protein extracted from synchronous *P. falciparum* culture. We observed the enrichment of the H3K64me3 mark in the ring and trophozoite stages, whereas a significant reduction of the H3K64me3 mark in the multinucleated schizont stage is observed (Fig. 5*A*). The immunoblot for H3 control protein both on the blot and off blot has remained the same throughout all three stages of *P. falciparum* (Fig. 5*A*).

Furthermore, we investigated the levels of other well-reported gene repressor methyl marks on H3 protein viz H3K9me3 and H3K36me3, and we did not observe any changes in their levels as *P. falciparum* develops inside RBCs (Fig. 5*A*). The relative quantification of the H3K64me3 mark intensity to control H3 has shown a 4- to 5-fold reduction of the H3K64me3 mark in the multinucleated schizont stage than ring and trophozoite stages of *P. falciparum* (Fig. 5*B*). To substantiate our findings on H3K64me3 dynamic deposition, we performed immunofluorescence staining of the ring, trophozoite, and schizont stages with anti-H3K64me3 antibody followed by fluorescent-labeled secondary antibody. The results confirmed the dramatic reduction in the distribution of the H3K64me3 mark in the multinucleated schizont stage, whereas the methyl mark was present in the ring and trophozoite stages (Fig. 5, *C* and *D* and Fig. S7).

The H3K64me3 localization experiment demonstrated distinct nuclear localization and enrichment as foci colocalizing with the nuclear counterstain, Hoechst. The immunostaining pattern for H3K64me3 in the trophozoite stage is very similar to H3K9me3, a well-known repressor methyl mark in *P. falciparum* (Fig. S8). Reduction of the H3K64me3 mark in the schizont stage is similar to that of hypomethylation or demethylation at H3K64 in *P. falciparum;* thus, such dynamics may regulate the schizont specific gene expression in the parasite. Taken together, this study supports the dynamics of the histone core methyl mark in various developmental stages of *P. falciparum* and such dynamic deposition of H3K64me3 in IDC stages may control the expression of stage-specific gene expression in *P. falciparum*.

### Stage-specific ChIP analysis identifies selective loss of H3K64me3 on the genes of exported proteins in schizonts of *P. falciparum*

Reduction of the H3K64me3 mark at the schizont stage permits to study the function of this methyl mark in *P. falciparum*. To address the function, we examined the distribution of the H3K64me3 mark in various genomic positions of all the three IDC stages of *P. falciparum*. We performed a ChIP assay with anti-H3K64me3 antibodies. The sheared chromatin was prepared from synchronous *P. falciparum* culture (Fig. S9*A*) and subjected to ChIP followed by library preparation from the ChIP DNA and was subjected to sequencing using the HiSeq Illumina platform. The qualities of the sequencing reads are very good to proceed with the genomic alignment (Fig. S9*B*).

**Figure 6:**
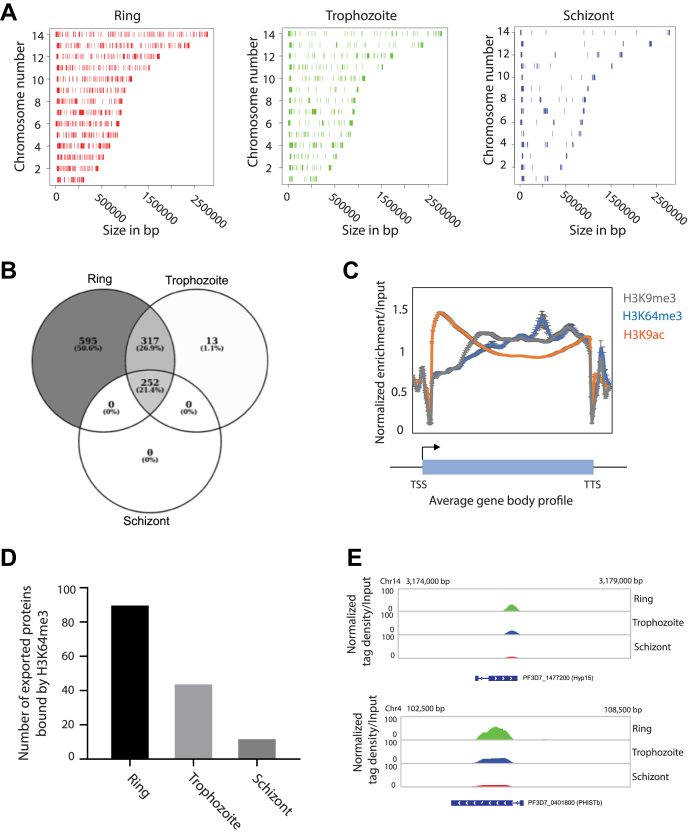
**H3K64me3, a repressive mark, is globally associated with the exported protein genes.** The ChIP sequencing identifies that H3K64me3 is specifically associated with the exported protein genes. *A*, binding sites of H3K64me3 identified during three different stages of the intra-erythrocytic developmental cycle (IDC) of *P. falciparum*. The occupancy of H3K64me3 is significantly decreased in multinucleated schizont stages. *B*, the Venn diagram showing the number of genes occupied by H3K64me3 during different stages of the IDC. There was a complete overlap of the genes occupied with H3K64me3 during the schizont stage with the ring stage–bound genes. *C*, the distribution profile of H3K64me3 over the average gene body of all the *P. falciparum* genes as compared with H3K9ac and H3K9me3, representative active and repressive histone modifications, respectively. Similar profiles were observed for both H3K9me3 and H3K64me3 histone modifications. *D*, the bar graph showing the number of gene-encoding exported proteins during three different stages of the IDC. H3K64me3 occupancy over exported proteins decreases as the stage progresses. *E*, IGV snapshot of H3K64me3 enrichment over two representatives of exported proteins. The decrease in the enrichment level can be observed during the trophozoite and schizont stages. IGV, Integrative Genomics Viewer; H3K64me3, trimethylation at lysine 64 on histone 3.

The H3K64me3-binding sites were determined after sequence alignment and normalization with input sequence. Interestingly, there was a significant reduction in the number of peaks on different chromosomes during the multinucleated schizont stage as compared with the ring and trophozoite stages (Figure 6, *A* and *B* and Fig. S10, *A* and *B*). These results were consistent with our previous results wherein we have observed a stage-specific immunoblot experiment that showed reduced deposition of H3K64me3 in the schizont stage (Fig. 5). Next, we looked at the average profile of H3K64me3 at the trophozoite stage across the gene body of all *P. falciparum* genes and compared it with the profiles of active (H3K9ac) and repressive (H3K9me3) histone modifications (Fig. 6*C* and Fig. S11, *A*–*C*). Interestingly, the H3K64me3 mark exhibits a distribution profile similar to repressive histone modification, H3K9me3. Moreover, a group of genes that code for export proteins were found to be enriched for the H3K64me3 mark during the ring stage of parasites, and there was a significant reduction in the total number of genes associated with the H3K64me3 as parasites progress into the trophozoite and schizont stages (Fig. 6*D*; Fig. S10, *A* and *B*). Furthermore, we validated the ChIP experiments with selected genes that code for export proteins and found a consistent reduction of the H3K64me3 mark on these in the schizont stage of *P. falciparum* (Fig. S11*D*) (Supplementary File 3). The reduction in the H3K64me3 mark is also shown on two representative export proteins, PF3D7_1477200 (Hyp15) and PF3D7_0401800 (PHISTb), using the Integrative Genomics Viewer (Fig. 6*E*).

### Association of the H3K64me3 mark with the subsets of exportome genes in *P. falciparum*

**Figure 7:**
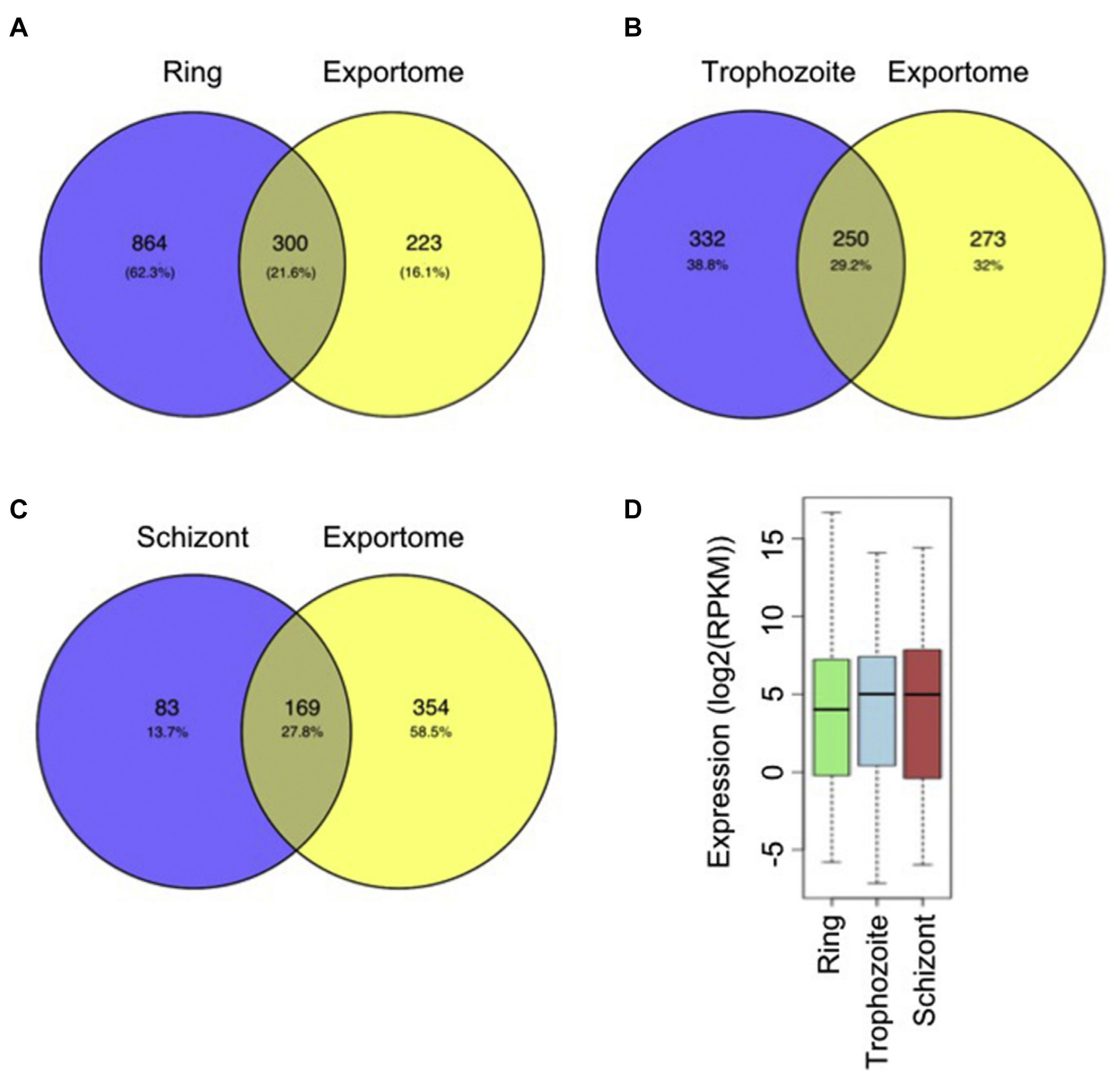
**Correlation of the H3K64me3 mark and exportome genes.***A–C*, the list of *Plasmodium* exportome is downloaded from the PlasmoDB for the ring, trophozoite, and schizont stages. Venn diagrams show the overlap between the *Plasmodium* exportome and H3K64me3 occupied genes in the ring, trophozoite, and schizont stages. The H3K64me3 mark occupies a significant number of exported protein genes during the ring stage and decreased when develops to multinucleated schizont stages. *D*, the box plot representing the average expression (log2(RPKM) of the genes occupied by H3K64me3 during the ring stage at three different stages of the *P. falciparum*. Upregulation in the expression of genes occupied by H3K64me3 during ring stages was observed as the parasite progress to the trophozoite and schizont stages. RPKM, reads per kilobase per million; H3K64me3, trimethylation at lysine 64 on histone 3.

As we observed enrichment of the H3K64me3 mark on a group of exported genes in the ring and trophozoite stages, to get the functional insights, we analyzed the gene list of *Plasmodium* exportome, which includes the list of exported proteins from different sources (PlasmoDB). The exportome genes that are associated with H3K64me3 during the ring stage are found to be 300 genes, which are associated with the H3K64me3 mark and are exported during the blood stage of the parasites (Fig. 7*A*). The total number of exportome genes occupied by the H3K64me3 mark is decreased when the parasite develops to the multinucleated schizont stages (Fig. 7, *B* and *C*). This further substantiates the H3K64me3 ChIP sequencing results that the dynamics of the H3K64me3 mark on the group of exportome genes in *P. falciparum* during its development in blood stages.

To understand the functional implications of H3K64me3 modifications on the exportome genes, we have used the published RNA sequencing data (GSE23865; 7) and examined the expression levels of genes to which the H3K64me3 mark was associated at the ring, trophozoite, and schizont stages. We have observed that the expression level of genes associated with the H3K64me3 mark at the ring stage increases as the parasite develops into the multinucleated schizont stages in which the mark is found to be reduced (Fig. 7*D*). Moreover, we confirmed the expression of the selected export genes by qRT-PCR and found upregulation of these genes in multinucleated schizont than ring and trophozoite stages (Fig. S12). This suggests that the H3K64me3 mark functions as a repressor methylation mark to a group of exportome genes during the ring and trophozoite stages and reduction of the H3K64me3 mark leads to the upregulation of these genes in schizonts. An earlier study on the stage-specific transcriptome analysis had shown increased levels of transcripts of an exported protein family of genes in the multinucleated schizont stage of *P. falciparum* (29), which may be due to the loss of the repressive H3K64me3 mark at that stage. Taken together, our data provide the evidence on the dynamics of the H3K64me3 mark on the subsets of genes encoding for exported proteins in *P. falciparum*.

## Discussion

The human malaria parasite adapts the complex life cycle to establish the infection in the host. The parasite undergoes massive transcriptional changes and chromatin modifications during the erythrocyte stages (20, 30, 31). It is known that the malaria parasite encodes a minimal number of transcription factors and the parasite's chromosomes are found to be dynamic (32, 33); however, very less is known about how the parasite controls stage-specific differential gene expression during its development in RBCs. Emerging reports have shown that the parasite largely depends on histone post-translational modifications for chromatin structure changes or the exchange of core histones in *P. falciparum* (34). In the present study, we report an unconventional trimethylation mark at K64 in the H3 core subunit, and the modification is dynamically distributed in the RBC stages of the malaria parasite. The H3K64me3 mark is located on the globular domain of the H3 subunit on its lateral side of histone octamers making direct DNA contact points in the nucleosome. We speculate that the dynamics of the H3K64me3 mark on histone core orchestrates the chromatin structure regulation during its IDC cycle and thereby regulates differential gene expression that helps the parasite to complete its life cycle inside host RBCs.

An earlier report on the H3K64me3 mark in mammals had shown that the methyl mark is associated with the short heterochromatin structure and functions as a repressor methyl mark (24, 25). However, the H3K64-specific methyltransferase was not identified so far, but it was reported that knockdown of Suv39H1, a well-known H3K9 methyltransferase, leads to the reduction of the H3K64me3 mark and could methylate both H3K9 and H3K64 positions in mammals because of conservation of the RK motif in both positions on the H3 substrate (24) (Fig. S4). In this study, we identified that *P. falciparum* evolved with two SET domain-containing methyltransferases that methylate at H3K64 (Fig. 2*B*). Moreover, our study identifies that the homolog of Suv39H1 in *P. falciparum,* that is, PfSET3, methylates only at the H3K9 position and does not methylate at H3K64 (Fig. 2*D*). In mammalian cells, it was proposed that establishment of the H3K64me3 mark is dependent on the presence of other well-known repressor methyl mark H3K9me3 (24). Also, in *P. falciparum,* we observed overlapping distribution profile for both H3K9me3 and H3K64me3 marks in our ChIP sequencing analysis (Fig. 6*B*). However, it is not known whether the H3K9me3 mark is essential for the methylation at H3K64 by PfSET4 and PfSET5 proteins in *P. falciparum*; thus, a detailed investigation is essential to address how these three proteins PfSET3, PfSET4, and PfSET5 are coordinated to introduce H3K9me3 and H364me3 marks in various genomic positions in *P. falciparum*.

The *P. falciparum* genome encodes 10 SET domain–containing proteins, and systematic gene KO studies of all SET proteins in *P. falciparum* revealed that PfSET4 and PfSET5 KO parasites are viable (35). However, we failed to obtain successful double KO for PfSET4 and PfSET5 genes in *P. falciparum*, which implies that the dynamic introduction of the H3K64me3 mark by these two SET enzymes might be essential for the growth of *P. falciparum* in its RBC life cycle. The PfSET4 and PfSET5 enzymes are unique among other histone lysine methyltransferases as these proteins strictly methylate the intact nucleosome substrate and do not methylate the free histone substrates (Fig. 2*E*). The structural data and molecular docking of the PfSET5 protein have identified that the PfSET5 protein forms the dimer and binds to the DNA of the nucleosome through its disordered basic amino acid patches. The PfSET4/PfSET5 enzymes prefer to methylate the nucleosome substrate and does not methylate free histone substrates; however, we also observed methylation with short peptides. The possible reason could be the peptides are short and small and therefore can easily get into the active sites of enzymes, whereas histone proteins (except the tail) have a folded globular structure larger than peptides that the target K64 site may not be easily accessible to the active site of PfSET4 and PfSET5 enzymes. Because the PfSET5 enzyme does not methylate the free histone substrates, PfSET5 protein may undergo conformational changes upon interaction with nucleosome substrate to reach the target K64, which resides underneath the DNA wrap on the histone core for its successful methylation. The methylation activity of the PfSET5 enzyme was decreased by changing the basic amino acid at the binding interface to acidic amino acid (K64E). A similar reduction of enzyme activity was obtained with the digestion of DNA on the nucleosome with DNase, which concludes the preference of nucleosome as the substrate by the PfSET5 enzyme.

**Figure 8:**
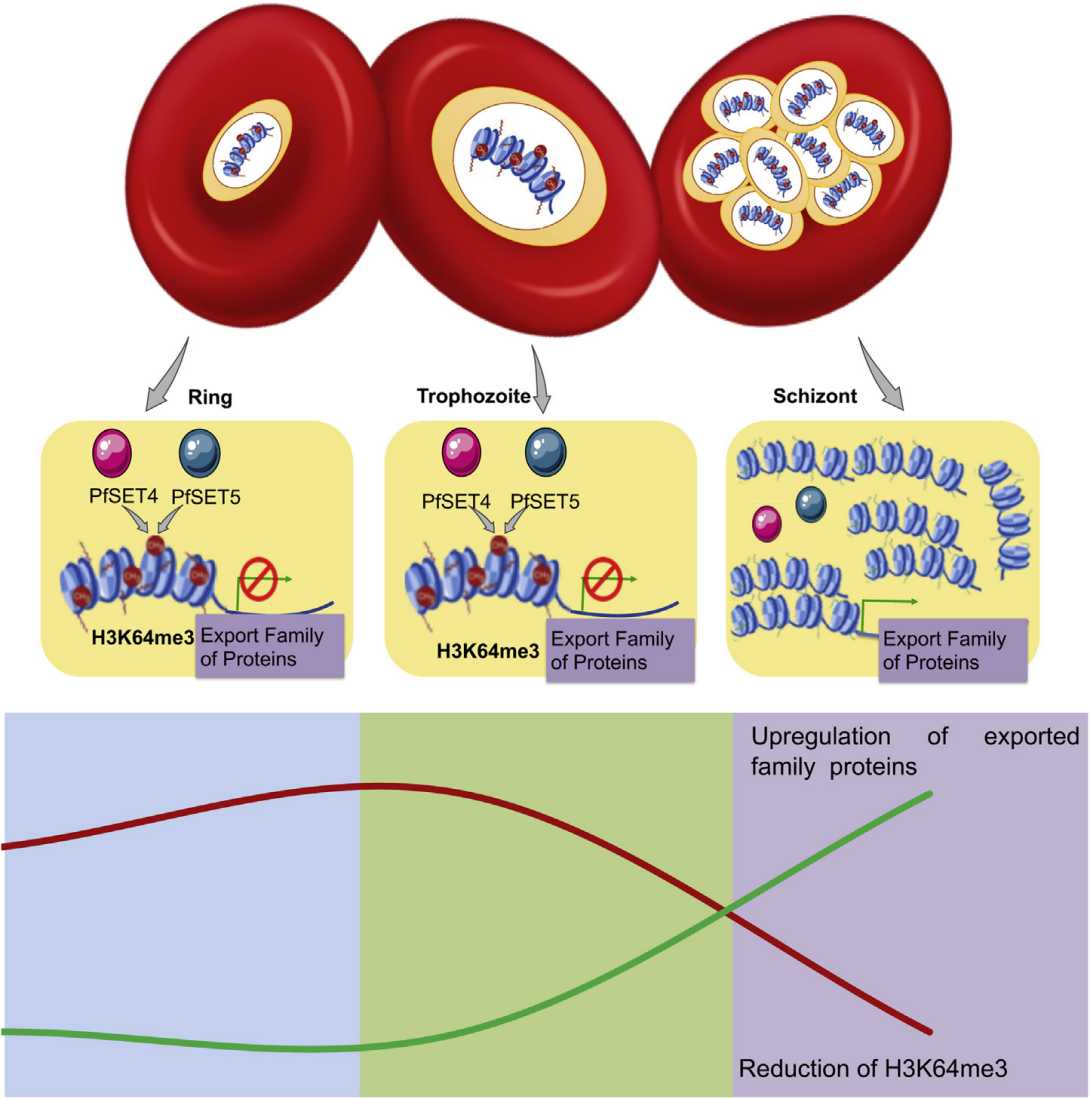
**The schematic model shows the presence of the H3K64me3 mark in ring and trophozoite stages, methylated by PfSET4 and PfSET5 enzymes, and the H3K64me3 mark is reduced in the multinucleated schizont stage of *P. falciparum*.** The proposed model for expressions shows reduced expression of exported family proteins in ring and trophozoite stages, and it is upregulated in the multinucleated schizont stage of *P. falciparum*. The *bottom* graph represents an inverse correlation of the H3K64me3 mark and expression of subsets of exported family proteins in RBC stages of *P. falciparum*. H3K64me3, trimethylation at lysine 64 on histone 3; RBCs, *red* blood cells.

The H3K64me3 mark is dynamic and is significantly reduced in the multinucleated schizont stages of *P. falciparum*; the reduction of the methyl mark in the schizont stage might be due to the presence of active H3K64me3-specific demethylases in the parasite. The stage-specific gene expression in human parasite *Plasmodium* spp. is still unclear. This study provides evidence on dynamics of unconventional epigenetic modifications, and these modifications might involve in the regulation of schizont-specific gene expression in *P. falciparum* (Fig. 8). Thus, a detailed biochemical analysis of PfSET4 and PfSET5 enzymes may pave the way for the exploration of the epigenetic enzymes as potential epidrug targets in the malaria parasites that demand further study.

## Experimental procedures

### *P. falciparum* 3D7 culture maintenance

Asexual blood stages of *P. falciparum* 3D7 strain was cultured at 5% hematocrit using advanced RPMI 1640 (# 12633-012, Gibco) supplemented with 2-mM L-glutamine (# 2530081, Gibco), 0.6% AlbuMAX II (# 11021-037, Gibco) and 10% human O+ Serum (36). Parasite cultures were then incubated at 37 °C, in the presence of B+ RBCs under a gas phase of 5% CO_2_. The cells were synchronized, and ring-stage parasites were selected by incubation with 5% D-Sorbitol (# S1876, Sigma) for 7 min as per the protocol (37). After confirmation of stages by Giemsa staining (# GS500, Sigma), the collected parasites were released from infected RBCs by lysis with 0.15% saponin (# 47036, Sigma). The lysed RBCs were pelleted at 14,000 RPM for 10 min at 4 °C to separate the parasites and were washed with ice-cold PBS for 3 times and used for histone extraction. The medium was prewarmed to 37 °C before usage, and the fresh medium was supplemented every alternate day to maintain the continuous culture. The growth of parasite was monitored by microscopy using standard Giemsa-stained blood smears.

### Histone extraction from *P. falciparum*

The parasites were lysed by mixing with the lysis buffer (10-mM Tris, pH 8, 1.5-mM MgCl_2_, 1-mM DTT, 100-mM NaCl, and eukaryotic protease inhibitor (# HY-K0011, MCE)) for 45 min at 4 °C. The lysate was then centrifuged at 10,000*g* for 10 min, and the supernatant was discarded to collect the pellet. The collected pellets were resuspended with 0.2 M H_2_SO_4_ and incubated for 2 h at 4 °C for acid extraction of basic histone proteins. This protein mixture was centrifuged for 10 min at 16,000*g*, the supernatant obtained was collected, and the histone proteins in this supernatant were precipitated by incubating with 33% trichloroacetic acid (Merck # 100807) overnight. The histone pellets were collected by centrifuging the precipitate at 16,000*g* for 10 min, and the histone pellets obtained were washed with ice-cold acetone. The final pellet was resuspended in sterile water after air-drying off acetone, and the concentration of proteins was measured at absorbance at 280 nm by nanodrop (NanoDrop 2000, Thermo). The quality of histone proteins was analyzed by separation on 16% SDS-PAGE and stained with Coomassie Blue.

### In-gel digestion

The histone gel bands were excised from the SDS-PAGE and placed on a clean glass plate. The gel bands were further cut into ∼1-mm square pieces with a sterile scalpel blade and were placed into a microcentrifuge tube. 300 μl of 50-mM ammonium bicarbonate/acetonitrile (1:1, vol/vol) was added to each tube and incubated with occasional vortex for 60 min, and the supernatant was discarded. To destain the gel pieces completely, 500 μl of acetonitrile was added and incubated at room temperature with occasional vortex, until gel pieces became white and shrunk. The supernatant was again removed. The gel pieces were saturated with sequence grade trypsin (13 ng/μl) (Sigma) in 50-mM ammonium bicarbonate. Enough trypsin was added to cover the dry gel pieces (∼35 μl) and was incubated on ice for 45 min. After the stipulated time ∼20 μl of 50-mM ammonium bicarbonate was added to cover the gel pieces completely and kept them wet during enzymatic digestion. Digestion was performed by placing the tubes with gel pieces in a thermostat and incubating the samples overnight at 37 °C. The extraction of the digested peptides was performed by adding 100 μl of the extraction buffer (1:2 (vol/vol) 5% formic acid/acetonitrile) to each tube and incubating for 20 min at 37 °C in a shaker. The supernatant was collected into a PCR tube and freeze-dried in a vacuum lyophilizer. To the freeze-dried product in the tube, 20 μl of 0.1% TFA/acetonitrile (vol/vol) was added and was centrifuged at 14,000 rpm for 12 min. The supernatant was collected into a PCR tube and further used for MALDI-TOF MS analysis

### Sample preparation and MS analysis of histone 3 protein

One part of the alpha cyano 4 hydroxycinnamic acid matrix saturated in TA50 (50:50 v/v acetonitrile: 0.1% TFA in water) was mixed with one part of the peptide sample solution, and the matrix/analyte mixture was spotted onto the polished steel MALDI-TOF target plate (Bruker Daltonics) and allowed to air dry (dried droplet method). The dried samples were analyzed using Ultraflextreme MALDI TOF/TOF (Bruker Daltonics) operated in a positive ion mode and controlled by a software program, FlexControl, version 3.3 (Bruker Daltonics). An average of 1500 laser shots was accumulated per spectrum in MS with a mass window of 700 to 3500 Da and 9500 laser shots for MS/MS (LIFT) mode. Machine parameters used were as follows: ion source I–25 KV, ion source II–22.25 KV, detector gain 8.5× for the MS mode, and ion source I–7.54 KV, ion source II–6.84 KV, LIFT I–18.99 KV, and LIFT II–2.85 KV for the MS/MS mode. Mass spectrum was calibrated using peptide calibration standard with a mass range of 700 to 3500 Da (Bruker Daltonics) for peptides, which includes bradykinin m/z 757.3992, angiotensin II m/z 1046.5418, angiotensin I m/z 1296.6848, substance P m/z 1347.7354, bombesin m/z 1619.8223, ACTH clip 1 to 17 m/z 2093.0862, ACTH clip 18 to 39 m/z 2465.1983, and somatostatin 28 m/z 3147.4710.

### MS data analysis

The acquired spectral data were analyzed using flexAnalysis, version 3.2 (Bruker) for identifying and annotating the peaks with a mass shift for monomethylation, dimethylation, or trimethylation. MS/MS data were then generated from the corresponding peptides having a mass shift for methylation using LIFT technology embedded in an Ultraflextreme MALDI-TOF mass spectrometer. The MS/MS peaks were then transferred to BioTools (version 3.2), an interactive analysis tool that helps extract in-depth spectral information and automatic annotation of spectra from the mass spectra generated. The search engine used was MASCOT (version 2.3, Matrix Science, London, UK) for protein identification. During database search, oxidation of methionine, trimethyl (K), trimethyl (R) was selected as a variable modification, and cysteine carbamidomethylation was selected as a fixed modification. Trypsin was chosen as the enzyme used with the specificity of one missed cleavage and peptide mass tolerance window of ±0.7 Da, fragment mass tolerance of ±1.2 Da. The database used was entries of PlasmoDB.

### Western blot analysis of histone methylation marks

The separated histone proteins in 16% gel were transferred on to PVDF membrane (0.2 μm, # BSP0161, PALL Life Sciences). The membrane was blocked in 5% skim milk, and primary antibodies against histone modifications were used at the dilution H3K64me3 (1:1000) (# C15410211, Diagenode), H3K9me3 (1:1000) (# C15410056-10, Diagenode), H3K36me3 (1:1000) (# C15410192, Diagenode), and H3 (1:3000) (# C15210011, Diagenode) and were incubated overnight at 4 °C. All these antibodies were generated against human histone peptide sequences. Horseradish peroxidase–conjugated secondary antibody (# sc-2030, goat anti-rabbit IgG-HRP, Santa Cruz) was used at 1:5000 dilutions against histone mark–specific primary antibodies and 1:8000 against H3-specific antibody. The blots were developed using Pierce ECL (# 32106, Thermo) Western blotting substrate.

### Cloning, expression, and purification of SET domains

The predicted SET domain constructs were made by PCR amplification from cDNA of the parasite using respective domain-specific primers (Supplementary File 2, sheet 1). The amplicons were then cloned into pGEX6P2 vector (GE Healthcare Life Sciences) using BamHI (NEB # R3136S) and XhoI (NEB # R0146S) restriction sites. All clones were sequenced to confirm the frame alignment. The SET domain protein was expressed in BL21-CodonPlus cells, which were grown till absorbance at 600 nm of 0.6 at 37 °C, and the protein expression was induced with 1-mM IPTG (# 16758-10, Sigma) and incubated further overnight with shaking at 18 °C to achieve the maximum expression level. The cells were collected and resuspended with the sonication buffer (20-mM Hepes buffer, pH 7.5, 500 mM KCl, 1 mM DTT, 1 mM EDTA and 10% glycerol), containing protease inhibitor cocktail (# P8849, Sigma) and lysed with a sonicator (Sonics Vibram-Cell) under 38% amplitude with pulse 1 s ON and 1 s OFF. The soluble fraction was collected by high-speed centrifugation at 12,000 rpm for 1 h and passed on to the Glutathione Sepharose High-Performance column (# 17-5279-01, GE Healthcare Life Sciences). The column was washed with the sonication buffer, and the bound proteins were eluted using the sonication buffer containing 40-mM reduced Glutathione (Sigma # G4251). The proteins were dialyzed against buffer I (20-mM Hepes, pH 7.5, 200-mM KCl, 1-mM DTT, 1-mM EDTA, and 10% glycerol) for 2 h at 4 °C and subsequently dialyzed against buffer II for 12 h (20-mM Hepes, pH 7.5, 200-mM KCl, 1-mM EDTA, 1-mM DTT, and 60% glycerol). The concentration of the proteins was measured using nanodrop, and purity was analyzed on an SDS-PAGE gel, stained with Coomassie Brilliant Blue stain, and the respective proteins were confirmed by Western blotting using an anti-GST antibody.

### Site-directed mutagenesis, expression, and purification of PfSET5 mutant proteins

A point mutation in the pGEX6P2 PfSET5 construct was generated by mega primer synthesis using the primer carrying the specific mutation (K64E, R73E, K114E) (Supplementary File 2, sheet 2), followed by rolling circle amplification. The WT plasmid was digested with DpnI enzyme (# R0176S, NEB) and transformed into XL1 blue cells to select the mutant transformants. The presence of the specific mutations in the plasmids was confirmed by Sanger sequencing (Fig. S6*A*). The PfSET5 mutant plasmids were transformed into BL21 strain, and proteins were expressed and purified using Glutathione Sepharose High-Performance column as described for PfSET domains purification. The quality of the proteins was analyzed on 12% SDS-PAGE gel and stained with Coomassie Blue.

### Histone and nucleosome substrate methylation assays for PfSET4 and PfSET5 proteins

The methylation activity for purified WT PfSET5 and mutant PfSET5 proteins was carried out with nucleosomes and histones as substrates isolated from *P. falciparum* 3D7 culture. The reaction mixture was prepared in the methylation buffer (250-mM Tris, pH 9, 20-mM DTT, 25-mM MgCl_2_), 4 μM of PfSET domain enzymes. The reaction was initiated by the addition of 0.76-μM tritium-labeled SAM (PerkinElmer) followed by incubation at 37 °C for 5 h. The reaction was terminated by using the gel-loading buffer (50-mM Tris, pH 6.8, 4% SDS, 12% glycerol, 2% β-mercaptoethanol, 0.01% Coomassie Brilliant Blue) and was heated at 95 °C for 15 min. The methylated full-length histones were then resolved on 16% SDS-PAGE gel. The gel was dried at 80 °C for 45 min and was exposed for 2 to 3 weeks at −80 °C with the X-ray sheets and was developed subsequently (38).

For immunoblot assays, the methylation activity for all purified SET enzymes (9 SET domains) was performed using 0.25 μM of nontritiated SAM (# A7007, Sigma) as a cofactor and synthetic mononucleosomes reconstituted with human histones proteins (# 16-0009, EpiCypher), which does not have any methylations as the substrate. The reaction mixture was prepared in the same methylation buffer and initiated by the addition of cold SAM (not radiolabeled) and incubated for 5 h at 37 °C. The reaction was terminated by using the gel loading buffer and was heated at 95 °C for 15 min. The methylated full-length histones were then resolved on 16% SDS-PAGE gel followed by immunoblotting with the anti-H3K64me3 antibody (1:1000 dilution) to identify the enzyme that methylates at the 64th lysine. The assay was repeated, and immunoblotting was performed with the H3K9me3 antibody to confirm the substrate specificity of the enzyme activity.

### Radioactive peptide methylation assay and any kD PAGE gel experiment

Synthetic H3K64 and H3K64A peptides were incubated with PfSET4 and PfSET5 proteins. The reaction mixture was prepared in the methylation buffer (250-mM Tris, pH 9, 20-mM DTT, 25-mM MgCl_2_), 150 μM of H3K64 WT and mutant peptides, and 4 μM of PfSET domain enzymes. The reaction was initiated by the addition of 0.76-μM tritium-labeled SAM (PerkinElmer) followed by incubation at 37 °C for 5 h. The reaction was terminated by using the gel loading buffer (50-mM Tris, pH 6.8, 4% SDS, 12% glycerol, 2% β-mercaptoethanol, 0.01% Coomassie Brilliant Blue) and was heated at 95 °C for 15 min. The methylated full-length histones were then resolved on any kD PAGE gel (# 1610144, Bio-Rad); it is a 40% acrylamide solution that permits to separate from 2-kDa protein to 150 kDa. The gel was dried at 80 °C for 45 min and was exposed for 2 to 3 weeks at −80 °C with the X-ray sheets and developed subsequently (38).

### Dot blot assay

The methylation activity of purified WT PfSET and mutant PfSET proteins was carried out with synthetic peptides (region spanning H3K9, H3K27, H3K36, H3K64, H3K64A) (Supplementary File 2, sheet 6). The reaction mixture was prepared in the methylation buffer (250-mM, Tris pH 9, 20-mM DTT, 25-mM MgCl_2_), 150 μM of peptides, and 4 μM of PfSET domain enzymes. The reaction was initiated by the addition of 0.25 μM of nonradiolabeled SAM (#A7007) followed by incubation at 37 °C for 5 h. After a brief cross-linking with the UV crosslinker (UVI Link CL-508), the blots were blocked in 5% skim milk and primary antibodies against histone modifications were used at the dilution H3K64me3 (1:1000) (# C15410211, Diagenode), H3K9me3 (1:1000) (# C15410056-10, Diagenode), and H3K36me3 (1:1000) (# C15410192, Diagenode) and were incubated overnight at 4 °C. Horseradish peroxidase–conjugated secondary antibody (# sc-2030, goat anti-rabbit IgG-HRP, Santa Cruz) was used at 1:5000 dilutions against histone mark–specific primary antibodies and 1:8000 against H3-specific antibody. The blots were developed using the Pierce ECL (# 32106, Thermo) Western blotting substrate.

### Surface electrostatic potential calculation

The available structure of the SET5 protein in the RCSB database (4RZ0) lacks the coordinates of disordered segments (residues 57–76 and 104–113) and modeled using the loop modeling routine in the MODELLER stand-alone package (39). Subsequently, the modeled structure is energy-minimized using GROMACS using AMBER99SB∗-ILDN force field to remove any steric clashes in the modeled structure (40). The surface electrostatic potential of the modeled structure is obtained by numerically solving the nonlinear Poisson–Boltzmann equation using PDB2PQR webserver at 310 K (41). The partial charges are assigned based on the AMBER force field, and the protonation states at pH 7 were determined using the PROPKA module (42, 43). The ionic strength conditions are simulated assuming a monovalent ion environment, and default dielectric constants (2 and 78.5 for protein interior and solvent, respectively) are used to scale electrostatic interactions.

### Immunofluorescence microscopy

Immunofluorescence staining was performed as described (44). Briefly, the smear of *P. falciparum*–infected RBCs made on glass slides were fixed in acetone:methanol (9:1) for 40 min at −20 °C. The slides were then washed with PBS and blocked with 3% bovine serum albumin in the presence of 0.01% saponin for better permeabilization for about an hour. The slides were then washed thrice in PBS for 5 min each. Primary antibody incubation was carried out for overnight at 4° C at following dilutions: H3K64me3 1:2000, 1: 3000, and 1: 5000 and for H3K9me3 1:3000; the slides were washed thrice in PBS. Fluorophore-conjugated secondary antibody was used at the following dilution: Alexa Fluor Plus 594 (#A21442, chicken anti-rabbit antibody, Invitrogen) of 1:500. The slides were washed thrice with PBS. Parasite nuclei were stained with Hoechst 333432 (# H3570, Invitrogen) at a dilution of 1:500 for 5 min. The slides were washed thrice with PBS and mounted using Fluoromount (# 00-4958-02, Invitrogen), and coverslips were placed. Images were collected using a confocal laser scanning microscope.

### Stage-specific ChIP sequencing for identification of the occupancy of H3K64me3 on a global scale

The *P. falciparum* 3D7 strain was cultured at 5% B+ve hematocrit in RPMI 1640 supplemented with 10% of O+ human plasma. The parasite was synchronized by 5% sorbitol treatment, and parasites were released from infected RBCs by lysis with 0.15% saponin. Crosslinked chromatin was prepared by adding 1% formaldehyde to the culture for 5 min followed by the addition of glycine to 0.125 M final concentration for 5 min to stop the cross-linking. Nuclei were isolated by homogenization in 20-mM Tris at pH 8.0, 6-mM MgCl_2_, and 0.4% Nonidet P-40 and collected on a 0.5 M sucrose-buffer cushion and suspended in SDS buffer (2% SDS, 100-mM Tris, pH 8.0, 20-mM EDTA, protease inhibitors). Chromatin was sheared by sonication in a Bioruptor UCD-200 (Diagenode) for 10 min at 30-s intervals, power setting high, to a size of 300 to 800 bp (34).

ChIP was performed by adding 1.8 μg of H3K64me3 antibodies, IgG (# C15 410206, Abcam) to ChIP buffer (100-mM NaCl, 20-mM Tris, pH 7.5, 6-mM EDTA, 1% Triton X-100). The three respective RBC stage samples of WT 3D7 were added to the mixture and incubated at 4 °C for overnight, followed by the addition of 15-μl protein A Dynabeads (# 10002D, Invitrogen) and further incubated for 4 h. After washing with buffers containing 180-mM NaCl, immunoprecipitated DNA was eluted and purified using PCR purification columns (MN-740609). Libraries for ChIP sequencing were prepared using the NEB Next Ultra II DNA library preparation kit. In brief, ChIP DNA and input DNA are subjected to various enzymatic steps to repair the ends and tailing with dA-tail followed by an adapter ligation. The adapter-ligated fragments are size-selected using SPRI beads and subjected to limited cycle PCR to generate the final libraries. The quality of the libraries was analyzed on Agilent TapeStation and then subjected to paired-end sequencing (50 cycles) using Illumina HiSeq platform.

### ChIP sequencing data processing and analysis

FastQC analysis was performed to determine the quality of sequencing reads, and adaptors were trimmed using Cutadapt. *P. falciparum* reference genome (PlasmoDB-37) was downloaded from PlasmoDB (http://plasmodb.org/plasmo/) and indexed using bowtie2 build. ChIP-seq data were mapped to indexed *P. falciparum* 3D7 genome using Bowtie2 with default parameters. The output from the bowtie2 aligner contains information regarding each read and its alignment to the genome. SAM output files from bowtie2 were converted into BAM files using SAMtools (45). Genomic regions associated with histone modification were identified using callpeak function of model-based analysis of ChIP-Seq (MACS2) with default parameters (--g size = 2.4e7, --nomodel, bandwidth, 300 bp; broad peaks, *p* value, 1.00e-5) (46). Input data were used as a control. Significantly enriched peaks (>log2 fold enrichment over input) were annotated using bedtools intersect (47). ChIP-seq signals were background-subtracted using MACS2 bdgcmp tool (deduct the noise by comparing two signal tracks in bedGraph) and were visualized using Integrative Genomics Viewer. Gene ontology analysis of the H3K64me3-associated genes was performed using PlasmoDB. The genes bound by H3K64me3 during different stages were checked for the export proteins. GraphPad Prism (version 8) was used for generating the bar graph representing the number of genes bound by H3K64me3. R script (http://r-project.org/) was used to visualize the H3K64me3 peaks chromosome wide at three different stages of *P. falciparum*. The average profile of histone modifications is calculated using seqMINER (48) by extracting the tag density in the gene body for all *P. falciparum* genes. Histone modification data for H3K9ac and H3K9me3 at the trophozoite stage were obtained from an earlier study (49). Data are normalized by dividing the reads per bin by total reads per modification.

### Correlation analysis of the H3K64me3 mark occupancy and gene expression

To determine whether H3K64me3 is associated with gene repression, we used published RNA sequencing data (GSE23865; 7) for three different stages of *Plasmodium* growth. The reads per kilobase per million values were calculated as described earlier (49). Expression analysis was performed at three different stages for the H3K64me3 mark–occupied genes at the ring stage. The box plot was generated using R box plot function with default parameters.

### Overlap in *Plasmodium* exportome and the H3K64me3 mark–occupied genes

The gene list of *Plasmodium* exportome is downloaded from the PlasmoDB (http://plasmodb.org). It included the list of exported proteins from different sources. The overlap between *Plasmodium* exportome and H3K64me3 mark–occupied genes at three different stages of *Plasmodium* growth was performed using the online tool Venny 2.1 (https://bioinfogp.cnb.csic.es/tools/venny/).

### RT-PCR analysis

Expression of selected export genes were analyzed by RT-PCR with RNA isolated from the ring, trophozoite, and schizont stages. The total RNA was isolated using the TRIzol method (50) followed by purification with RNeasy kit (# 74104, Qiagen). The eluted RNA was used for cDNA synthesis. The cDNA synthesis was carried out using the Maxima H minus First-strand synthesis kit (Cat # K1681, Thermo Scientific). Afterward, cDNA synthesis for all the reactions was carried out by PCR using following conditions: 25 °C for 10 min followed by 50 °C for 15 min; reactions were terminated at 85 °C for 5 min. The qRT-PCR was carried out with freshly synthesized cDNA using SYBR Green mix (Thermo Scientific) (51).

### Declaration

All the experimental studies abide to the Helsinki principles.

## Ethics statement

Human red blood cells used in this study were collected from healthy volunteers' blood samples, with due approval from the institutional (Rajiv Gandhi Centre for Biotechnology) ethics committee (IHEC/01/2014/05). Healthy volunteers provided written consent for the collection of blood to obtain the RBCs from healthy volunteers at the Rajiv Gandhi Centre for Biotechnology Molecular Diagnostic facility.

## Data availability

All the data generated or analyzed during this study are included in this published article and its supporting information files. All the Next Generation Sequencing files generated in this study are available for download from NCBI GEO accession number: GSE154651. The raw mass spectrometry data are deposited in figshare portal (accession # 10.6084/m9.figshare.14152973).

## Supporting information

This article contains supporting information.

## Conflict of interest

The authors declare that they have no conflicts of interest with the contents of this article.
