# Supplementary material for: Dynamic association of the H3K64 trimethylation mark with genes encoding exported proteins in Plasmodium falciparum
Source: J Biol Chem. 2021 Apr 9;296:100614. doi: 10.1016/j.jbc.2021.100614 (PMC8095176; doi:10.1016/j.jbc.2021.100614)
Supplement: Figures S1 to S12 [file mmc1.docx]

**Supplementary information for**

**Dynamic association of the H3K64 trimethylation mark with genes encoding exported proteins in *Plasmodium falciparum***

CA Jabeena^1,5^, Gayathri Govindaraju^1,5^, Mukul Rawat^2^, Soundhararajan Gopi^3^, Devadathan Valiyamangalath Sethumadhavan^1,5^, Abdul Jaleel^4^, Dhakshmi Sasankan^1^, Krishanpal Karmodiya^2^, Arumugam Rajavelu^1^*

**Supplementary figure 1**: **(A)** The table represents the list of peptides identified in *P. falciparum* H3 protein. The H3K64 peptide is highlighted in green color. The methylated target residue is underlined. **(B)** MS fragmentation spectra for KLPFQR modified peptide (832.292) equivalent to an additional mass of three methyl groups (Spectra represented in pink color) and unmodified peptides (788.53) (Spectra represented in blue color).

**Supplementary figure 2**: Dot blot assay with 1 nmoles of H3K9m3 and H3K36me3 peptides were spotted on the membrane. The PfSET5 methylation was performed with 150 µM H3K64 and H3K64A peptides and spotted on the membrane. The immunoblot assay confirms the methyl modification specificity for each antibody that were used in this study. The bottom images represent the Ponceau S staining of peptides on the membrane.

**Supplementary figure 3:** **(A)** Schematic representation of SET domain containing proteins annotated in *P. falciparum* database (PlasmoDB). The SET domain (pink color), and other regulatory domains like PHD (Blue colour) and Bromo (Red colour) are marked. The total numbers of amino acids are provided for each SET domain proteins. **(B**) The 9 SET domain proteins are successfully expressed and purified as GST tagged recombinant proteins, separated on 12% SDS-PAGE gel electrophoresis and stained with Coomassie brilliant blue. The SET domain proteins are marked with blue colour star. **(C)** Quality of the synthetic mononucleosomes (SM) was verified on 16% SDS-PAGE gel and stained with Coomassie brilliant blue.

**Supplementary figure 4**: Sequence alignment of *P. falciparum* and Human histone 3 proteins, the red box represents the DNA contact points of the histone 3 core in the nucleosome (Silberhorn et al., 2016). Blue color underlines represent the conservation of RK motif at the K9 and K64 positions in H3 protein, the red color asterisk represents the target lysine 64 (K64) for trimethylation.

**Supplementary figure 5:** Immunoblot assay with anti-H3 antibody confirms the presence of H3 protein in GST-H3 and GST cleaved H3 preparation that were used for *in vitro* methylation assay.

**Supplementary figure 6**: Expression and purification of PfSET5 and Pf SET5 K64E, PfSET5 R73E and PfSET5 K114E mutant proteins, separated on 12% SDS-PAGE gel and stained with Coomassie brilliant blue. The protein marked with red color asterisks, and the GST marked with black color triangle. The PfSET5 K114E mutant protein runs slight above to the expected size.

**Supplementary figure 7:** Additional representative immunolocalization images show that localization of H3K64me3 in ring and trophozoite stages and reduced Immunostaining in multinucleated schizont stage of *P. falciparum*. The represented scale bar is 5 µ.

**Supplementary figure 8:** Immunostaining of H3K9me3 shows typical punctuate nucleate localization at trophozoite stage of *P. falciparum*. The represented scale bar is 5 µ.

**Supplementary figure 9**: **(A)** Sheared chromatin (250 – 500 bp) prepared from ring (R), trophozoite (T) and schizont (S) stages of *P. falciparum* and size of the sheared chromatin was analyzed on 1.5% agarose gel stained with gel red. **(B)** Quality controls analysis of ChIP sequencing reads for trophozoite sample are provided and the quality data is very similar for ring and schizont samples.

**Supplementary figure 10**: **(A)** Table showing total number of peaks, significant peaks (> 2-fold enrichment) and corresponding genes occupied by H3K64me3 during three different stages of the intraerythrocytic development of the life cycle of *P. falciparum*. (B) Venn diagram showing number of genes occupied by H3K64me3 during three different stages of the intraerythrocytic development. Gene ontology for the genes associated exclusively during ring stage, during ring and trophozoite stage and during all three stages of the intraerythrocytic development of the *Plasmodium*.

**Supplementary figure 11**: **(A)** Heat map showing the ChIP-seq tag counts at 1177 H3K64me3 occupied *P. falciparum* genes across three different stages (1164 genes from the ring stage and 13 genes exclusively from the trophozoite stage) for H3K64me3 along with activation (H3K9ac) and repressive (H3K9me3) marks. The H3K64me3 mark was found to be enriched mostly at the 3’ end of the genes and towards the centre of the genes. **(B)** The heat map represents the ChIP-seq tag counts at 5685 *P. falciparum* genes for H3K9me3 and H3K9ac modifications. The H3K9me3 is only associated with a handful of genes on sub-telomeric sites. **(C)** Average distribution of the H3K9ac and H3K9me3 modifications over the gene body. The H3K9ac mark is distributed along the entire gene body, however the H3K9me3 mark is found more towards the centre and 3’ end of the gene body in *P. falciparum.* (**D**) Validation of H3K64me3 mark ChIP sequencing with selected target genes by ChIP qPCR (**Supplementary file 2, sheet 3**). The ChIP experiments were performed in triplicates and enrichment of the targets were normalized to input. The error bar represents the standard deviation of three independent experiments.

**

**Supplementary figure 12:** Relative expression of selected exported family genes from ChIP sequencing was measured using qRT-PCR with RNA isolated from a highly synchronous culture of all three stages of *P. falciparum.* The relative mRNA expression of these genes was normalized to the Seryl-tRNA synthetase gene of *P. falciparum*. An average ct value from technical replicates was calculated then average values were calculated from three independent biological replicates, the error represents the standard deviation (n=3).
